# Towards open-ended evolution in self-replicating molecular systems

**DOI:** 10.3762/bjoc.13.118

**Published:** 2017-06-21

**Authors:** Herman Duim, Sijbren Otto

**Affiliations:** 1 Zernike Institute for Advanced Materials, University of Groningen, Nijenborgh 4, 9747 AG Groningen, The Netherlands; 2 Stratingh Institute for Chemistry, University of Groningen, Nijenborgh 4, 9747 AG Groningen, The Netherlands

**Keywords:** autocatalysis, open-ended evolution, origin of life, self-replication, synthetic life

## Abstract

In this review we discuss systems of self-replicating molecules in the context of the origin of life and the synthesis of de novo life. One of the important aspects of life is the ability to reproduce and evolve continuously. In this review we consider some of the prerequisites for obtaining unbounded evolution of self-replicating molecules and describe some recent advances in this field. While evolution experiments involving self-replicating molecules have shown promising results, true open-ended evolution has not been realized so far. A full understanding of the requirements for open-ended evolution would provide a better understanding of how life could have emerged from molecular building blocks and what is needed to create a minimal form of life in the laboratory.

## Introduction

Mankind has always pondered upon its own existence and has sought to understand the origin of life. This led us to trace back our roots, from the great apes to a last universal common ancestor, a simple cellular lifeform from which all other present-day organisms have descended. Ultimately this leads us to one of the great questions in science; how can life emerge from inanimate matter? And even more interestingly, can we achieve such a process in the lab and create life from scratch?

There are many different theories surrounding the origin of life and several attempts have been made to realize the synthesis of de novo life. All theories involve the presence of molecules that can create copies of themselves at some stage. It remains unclear whether such molecules were already important at the very early stages of the origin of life or whether life started with large autocatalytic networks [1] and specific molecules that store genetic information only appeared later. These self-replicating molecules carry hereditary information in the form of their molecular structure that can be passed on to successive generations. If mutations occur during the replication process, genetic information can change from one generation to the next. Natural selection can act on these variations, favoring those varieties that are beneficial for the stability and reproduction of the replicator. Under the right conditions, such Darwinian type evolution can eventually lead to diversification and complexification of the molecules in the system.

This review aims to provide an insight into the historical background and recent developments in the field of in vitro evolution of self-replicating molecules. To do so, we will first cover a few important principles of Darwinian evolution and will show how these concepts apply to the case of molecular self-replication. This is followed by a description of some self-replicating systems and their properties, starting from the very first report on self-replication to more elaborate systems. Finally, some recent experiments concerning in vitro evolution of self-replicating molecules and networks will be discussed. We argue that, although systems that show intriguing evolutionary capabilities have been devised, there is still a long way to go before a system that is capable of true undirected or open-ended evolution has been realized. Worryingly, the phenomenon of open-ended evolution in itself is currently not well-defined nor understood. If we are to create life in the lab, a thorough knowledge of this concept and its prerequisites is probably essential.

## Review

### 1 Requirements for Darwinian evolution

One of the most remarkable and key features of life is the fact that it has a strong tendency (or, at least ability) to diversify and increase in complexity. Whereas life once must have started out as a comparatively simple and primitive form, it has diversified into a vast variety of species ranging from aquatic to airborne ones. The principles governing this diversification in biological systems were already described by Darwin in his famous work On the Origin of Species, but are still not understood in full detail [2]. It was only in the 1960’s that Spiegelman extended the scope of Darwinian evolution to chemical systems by studying the evolution of RNA-complexes [3]. In these experiments RNA was replicated using enzymes “borrowed” from contemporary biology. The outcome of the selection experiments was the shortening of the RNA sequence, as shorter sequences could be replicated faster. It was soon realized that a better understanding about how evolution acts on the molecular level would not only provide valuable insights into the origin of life and the emergence of species, but it could also pave the way towards the realization of synthetic life.

**Figure 1:**
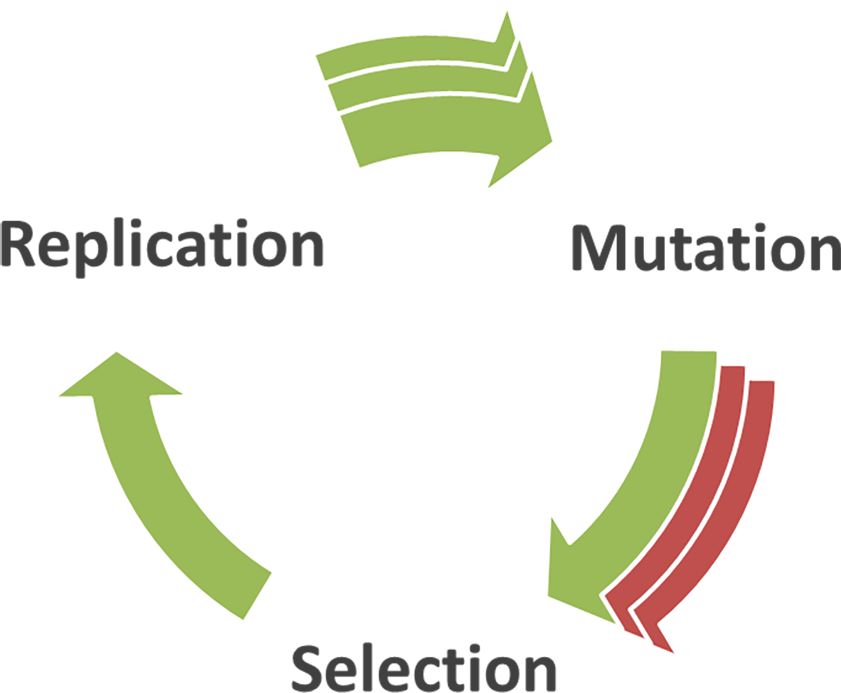
Three processes involved in Darwinian evolution. Species must be replicated to obtain a large population. During the replication process mutations can occur, on which natural or artificial selection can then take place.

In biology, Darwinian evolution in a chemical system can be considered to be the result of an interplay of the three different processes that are summarized in Figure 1 [4]. These concepts can, in principle, be extended to what we will consider as Darwinian evolution in chemical systems. First the parent molecule, or replicator, is replicated to yield a large number of copies. This can for instance be achieved via an autocatalytic cycle, as will be discussed below. Mutation involves the emergence of a difference between the parent template and its copies. The accuracy of the replication process of DNA is generally safeguarded by sophisticated enzymes, but systems that lack such machinery are more prone to occasional errors during replication. Mutations, however, might actually be advantageous for the replicator if the newly formed copies are more stable, replicate more efficiently or prevail under a change of environment. If such advantageous mutations arise, competition between different replicators might occur, leading to a process of natural selection and survival of the fittest replicator. We consider replication, mutation and selection to be necessary and sufficient conditions for Darwinian evolution.

#### 1.1 Replication

During replication a large number of copies of the replicator is produced via an autocatalytic process. In animals or other lifeforms it is quite clear that reproduction leads to a transfer of genetic information from the parent to the offspring. Not only are the vital structures of the organism transferred but also some peculiarities like a specific eye color or a hereditary disease is passed on to the next generation. The transfer of information in replicating molecules may be less obvious, but if one considers a polymer with a specific sequence of subunits, it is clear that some form of genetic information is transferred if the copies have an identical sequence to the parent molecule.

The survival of a particular molecular structure under a set of environmental conditions depends on both the rate of replication and the rate of decomposition of this replicator. If a replicator decomposes at a higher rate than that it is produced, that particular replicator may become extinct. If, on the other hand, the sequence and structure of the molecule is such that the replication rate exceeds its destruction rate, the replicator is sufficiently adapted to its environment and will persist under the given conditions.

#### 1.2 Mutation

The environmental conditions, to which a set of replicators is exposed however, may not be in a steady-state. Instead, the environment may be continuously changing. Consider for instance changes in temperature, acidity, light intensity and humidity to which a system will inevitably be subjected. A replicator that is very well adapted to a certain environment, might not persist at a later time when the environmental conditions have changed. In fact, if it were not for the presence of small mutations in the genetic information, a species would be very fragile. If the number of mutations is only small compared to the length of the molecule, hereditary information is still largely preserved. Moreover, the mutants may be better adapted to the new environmental conditions than their predecessors. If the mutants indeed have a higher rate of accumulation than their parents, they will eventually overtake their parents and will become the new dominant species. In practice a replicator generally has to exhibit exponential growth in order to dominate over a weaker replicator [5–7].

Eigen et al. noted, however, that the case is somewhat more complicated than a single type of mutant replicator overtaking another replicator. They introduced the concept of quasi-species, as an analogue to conventional species in biology [8]. A quasi-species consists of a master sequence with a dynamic distribution of closely related mutants. This concept captures the fact that for relatively high mutation rates not a single fittest replicator, but rather a distribution of closely related mutants survives. The mutants in this distribution around a master sequence all replicate at a different rate and are cross-catalytic, which leads to the production of further mutants. Selection in these systems thus does not act on the level of individual mutants, but rather on the entire quasi-species [8–10]. Such quasi-species behavior was recently reported in an in vitro evolution experiment with replicating RNA species [11].

There is of course a constraint on the number of mutations that can occur without losing too much hereditary information from the parent molecules. In the same work, Eigen showed that unless mutation rates were significantly diminished, the increase in the length of the genome would unavoidably lead to a catastrophic loss of hereditary information. That is, the replication process of a long molecule requires a much higher fidelity than that of a smaller molecule. If the rate at which errors in the replication occur exceeds a certain error threshold, the genetic information will disintegrate and the species will go extinct [8,12]. In fact, the reason that viruses are so good in adapting to different environments and always seem to be one step ahead of the defense mechanisms of the host is because the replication process of the viral genome operates very close to the error threshold, allowing for as many mutations as possible without the loss of genetic information [12–13].

#### 1.3 Selection

In biology natural selection operates on the phenotype, i.e., the observable traits of a species. An individual that is better adapted to its environment is more likely to survive then one that is less adapted. This higher survival rate will lead to a larger amount of offspring for that type of individual, favoring their presence in the population. The phenomenon of natural selection can also operate at the molecular level. This requires experiments to be run under conditions where replication and replicator destruction occur in parallel. Such conditions were employed in only a small subset of the work on self-replicating molecules where the emphasis has mostly been on replication in the absence of destruction. Which replicators then end up being selected depends on their rates of replication relative to their rate of destruction, or, as proposed by Pross, their dynamic kinetic stability [14]. Selection in the Darwinian sense requires extinction of the weaker replicators, so that only the fitter ones remain. There are some detailed kinetic considerations that lead to specific mechanistic requirements for the replication process. Szathmary and Lifson showed that in a scenario where different replicators compete for common building blocks, extinction of the weakest replicators occurs only if the kinetic order of the replicator in the replication process is at least equal to the order of the replicator in the destruction process [5–6]. As for most plausible mechanisms the destruction process is first order in replicator, this implies that the replication process must also be at the least first order in replicator; i.e., replicators need to be able to grow exponentially in order to exhibit Darwinian evolution in the most common scenarios. This consideration has spurred many efforts to develop exponential replicators, which are far from trivial to produce (vide infra). But even with exponential replicators, Darwinian evolution does not necessarily lead to complexification and the spontaneous emergence of new function, as the Spiegelman experiments made painfully clear [3].

Yet, in order to obtain a form of life from a molecular system, it must be able to grow increasingly complex and diverse. Systems that undergo such undirected diversification may in the end give rise to ecosystems full of complex organisms or structures [15]. Note that these organisms then would all be part of an evolving ecosystem, and it has been argued that a proper and complete description of life should therefore not only be at the individual level but also at the level of entire ecosystems [16].

#### 1.4 Dynamic kinetic stability

Pross has introduced the useful concept of dynamic kinetic stability for describing the fate of systems in which replication and selection occur concurrently [14]. The idea is that the stability of a self-replicator in a system in which replication and destruction processes occur simultaneously is not determined by the thermodynamic stability of the replicator, nor by the rate of formation of the replicator alone, but by the balance between the rate of formation and the rate of destruction of the replicator. As either replication or destruction (or both) are typically coupled to other chemical reactions that convert high-energy reactants to low-energy products, replication in a replication/destruction regime should normally be chemically fueled. Such fueling, in principle, allows complexification of the replicator, without defying the second law of thermodynamics, as the system as a whole still evolves towards increasing entropy. With replicator complexification having been made feasible, it then only depends on evolutionary possibilities and benefits whether complexification also actually occurs.

In order for the considerations of dynamic kinetic stability to apply and in order for Darwinian evolution to occur, it is essential that replicators are subjected to a replication–destruction regime. Unfortunately, until now, very few systems reported in the literature are (see below).

#### 1.5 Open-ended evolution

As mentioned before, the Darwinian triad of replication, selection, and mutation in itself is not sufficient to drive the complexification of a chemical system. But what determines whether a (chemical) system is capable of growing in complexity or is condemned to remain at a low level of complexity? This is a question that is not only relevant in evolutionary chemistry, but also has far-reaching consequences for the development of artificial life in computer models. As Moreno and Ruiz-Mirazo point out, in order for a system to fully evolve it should not only exhibit structural variety, but also some form of functional variety [17]. In the context of this review, we consider such function as any property of the replicator that benefits the dynamic kinetic stability of the system as a whole. A term that is widely used to describe the emergence of novel functionality is that of open-ended evolution. Although a clear consensus about a definition of open-ended evolution is lacking in literature, we will adopt the definition provided by Taylor here. Open-endedness means *the capability of components in a system to develop new forms continuously* [18]*.* From this definition it follows that a self-replicating system should be able to explore a huge number of possible mutants, otherwise the system will either get trapped in a stationary optimum situation or will recycle already explored forms of the replicator [19–20]. Both of these situations cannot lead to the continuous development of new forms of replicators and are thus detrimental to the open-endedness of the system. Another requirement is that the total structural space available to the system should exceed by many orders of magnitude the actual structural space that the system occupies at any one time, or as Maynard-Smith and Szathmáry put it; the replicators should possess unlimited heredity [21]. It is also important to note that newly evolved replicators are not necessarily more advanced or better than the original replicator. It is the mere development of novelty that is the vital aspect of open-ended evolution, causing it to be an undirected process that does not necessarily entail progress [18].

It is however not that trivial that a replicator can give rise to such a large number of new forms. As Crutchfield and Schuster pointed out, the dichotomy of genotype and phenotype is a powerful mechanism to obtain such a vast number of possible mutants [22]. Since mutations act on the genotype only and selection pressure exclusively acts on the phenotype, the two mechanisms are partially decoupled. If this were not the case only those mutations that are favored by selection will occur, strongly decreasing the possible number and randomness of new forms of the replicator.

It is apparent that open-ended evolution plays an important role in the emergence of novelty from simple replicators and that Darwinian evolution alone is an insufficient requirement for true unbounded evolution in a chemical system. This undirectional evolutionary process is therefore considered to be of importance in the transition from inanimate matter to life. The exact principles governing open-ended evolution are however not yet fully understood and it is not clear what the precise requirements for a system are in order for it to be capable of open-endedness [23].

In the following section we will discuss some basic principles of self-replication, followed by a discussion on recent developments towards the realization of open-ended evolution in chemistry.

### 2 Replicating systems

The most instructive and intuitive self-replicating system to consider is probably that of DNA. A DNA molecule consists of two strands of nucleotides that are intertwined to form a double helix. During the replication process of DNA, each of these strands can act as a template for the formation of a complementary strand. In this way an exact copy of the original structure of DNA is formed and the DNA has successfully become replicated. The replication of DNA however is a complex process mediated by enzymes such as DNA polymerase and topoisomerase. To better understand the origin of life and as a possible first step in the synthesis of de novo life it would be very interesting indeed to achieve molecular replication without the need of such enzymes, since enzymes themselves must be products of an evolutionary process and can thus not explain the emergence of living systems from basic chemical building blocks. The following section will treat a representative selection of self-replicating systems, for a more comprehensive overview, see: Philp and Vidonne [24], Von Kiedrowski and Bag [25] and Bissette and Fletcher [26].

#### 2.1 Minimal self-replicating system

**Figure 2:**
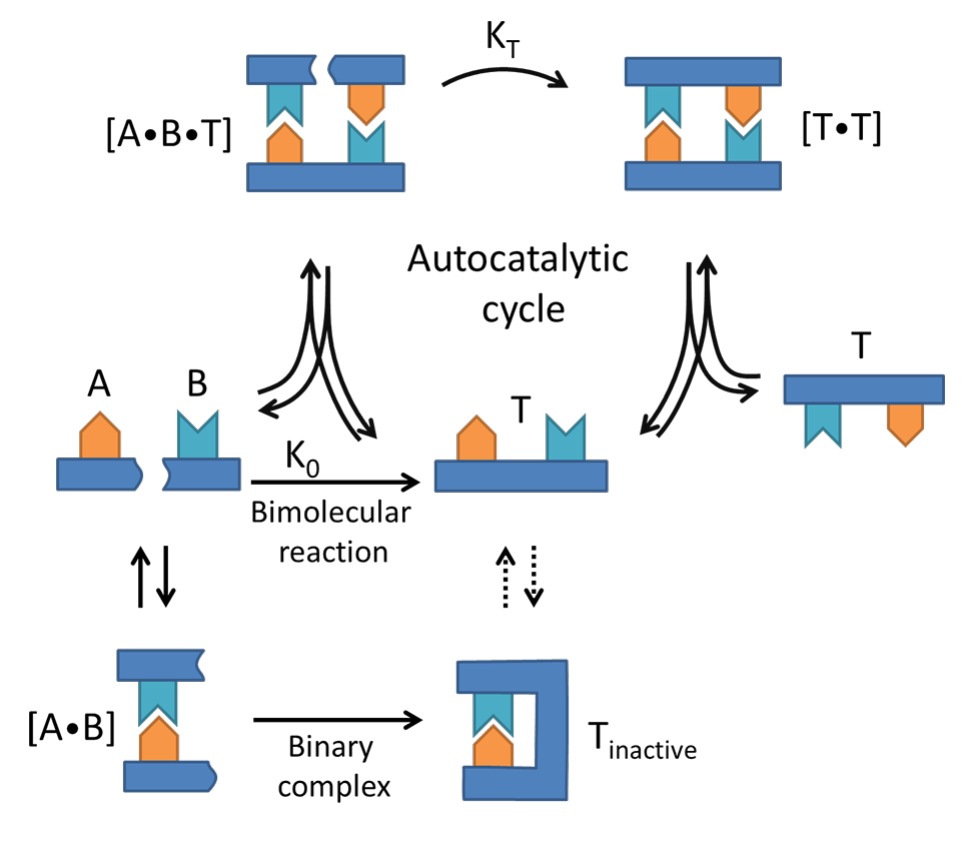
Minimal system for self-replication. Building blocks **A** and **B** can react to form either template **T** or its inactive counterpart **T_inactive_**. The formation of template **T** can direct **A** and **B** to a configuration in which they are in close proximity, accelerating the reaction between **A** and **B** leading to the formation of the [**T∙T**] complex. Dissociation of this complex completes the replication cycle of the initial template molecule.

The simplest form of a self-replicating system is that in which the replicator acts as a catalyst for its own formation from a set of basic building blocks. This fundamental form of a self-replicating system is depicted in Figure 2 and is called a minimal self-replicating system. An essential requirement for a minimal replicating system is that molecules **A** and **B** are complementary to template **T** so that they are able to bind to it via noncovalent interactions.

Figure 2 shows three different channels in a minimal replicating system. Building blocks **A** and **B** can react via the bimolecular reaction pathway, to form the template molecule **T**. In the second pathway – binary complex formation – **A** and **B** bind together reversibly to form a complex [**A∙B**]. This complex may undergo a covalent reaction if **A** and **B** experience an increased effective molarity, leading to an inactive template **T_inactive_** which is folded back onto itself. The third pathway in the minimal replication system is the autocatalytic cycle. In this cycle, the building blocks **A** and **B** bind reversibly to the complementary recognition sites on the template molecule **T**. This arrangement brings molecules **A** and **B** in close proximity, leading to an increased effective molarity and enhanced rate of bond formation. When **A** and **B** ligate to each other, a [**T∙T**] complex is formed, which can then dissociate to yield two identical **T** molecules. The autocatalytic cycle thus leads to a replication of the original template molecule. The final, and often overlooked, pathway was identified by Reinhoudt et al. following a fierce discussion between Rebek and Menger about the mechanisms involved in the self-replication in their systems [27]. In this pathway (not depicted in the diagram) one of the building blocks, say **A**, binds to the template molecule. In certain systems this can lead to the activation of **A**, such that **B** can then react with **A** directly from solution.

Initially, when there are virtually no template molecules in the mixture but only building blocks **A** and **B**, the bimolecular and binary complex pathways leading to the formation of **T** and **T_inactive_** will be dominant. Clearly, the inactive template cannot lead to autocatalysis and therefore hinders the self-replication process. Upon formation of **T**, the autocatalytic pathway will become increasingly important, in principle allowing for exponential growth of the template. A requirement for effective autocatalysis, however, is the dissociation of the [**T∙T**] complex into two individual template molecules. If this complex does not dissociate, the newly formed template molecule cannot lead to further enhancement of the reaction rate, effectively arresting the autocatalytic cycle. Such product inhibition is an important limiting factor in many synthetic replicator systems and prevents them from attaining exponential growth.

#### 2.2 Reciprocal self-replicating system

**Figure 3:**
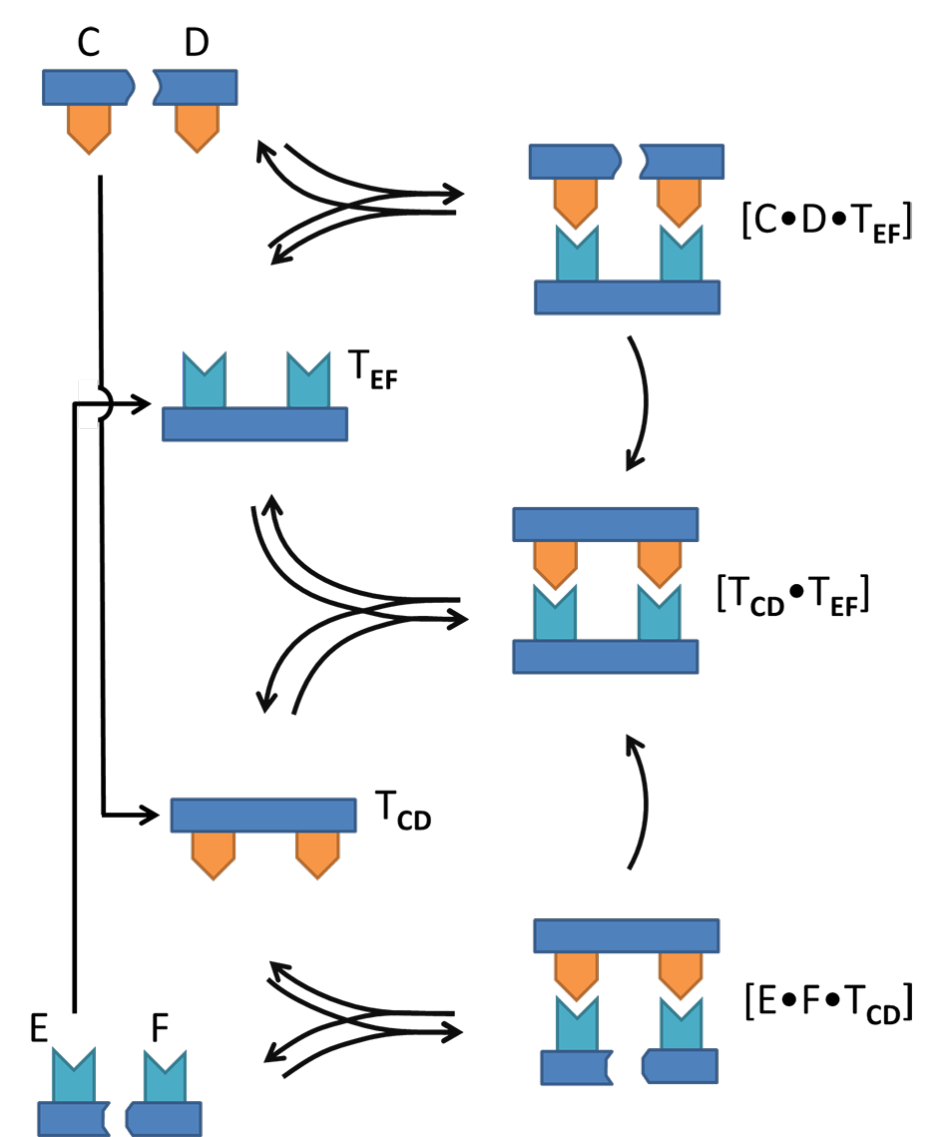
A cross-catalytic replication scheme in which the formation of one template stimulates the formation of a different, complementary template.

A more complicated situation arises when the template molecules under consideration are no longer self-complementary, but instead are complimentary to a second template molecule. The replication of DNA is a prime example of such a reciprocal self-replicating system. One strand of the double helix acts as a template for the formation of the other complementary strand and vice versa. Figure 3 shows a schematic representation of a reciprocal replicating system. It consists of two catalytic cycles which both lead to the same template duplex [**T_CD_∙T_EF_**]. Instead of only two building blocks the reciprocal system has four basic building blocks labeled **C**, **D**, **E** and **F**. Building blocks **C** and **D**, can react to form the template **T_CD_** which catalyzes the formation of the complementary template **T_EF_** from building blocks **E** and **F**. Similarly the **T_EF_** template can promote the formation of the **T_CD_** template.

#### 2.3 Reaction kinetics and its implications

When considering a mixture containing only building blocks **A** and **B** in the minimal replicator model (Figure 2), the formation of template molecules **T** can initially only take place via the bimolecular reaction pathway. The bimolecular reaction is a relatively slow reaction, since it involves the unassisted formation of a covalent bond between the two reactants. However, if a sufficiently large amount of the template molecules is formed, the autocatalytic cycle will play an increasingly dominant role. Because the catalytic cycle leads to a doubling of the template molecules after each run, an exponential increase in the concentration of the reaction product would be expected. Naturally, this exponential increase cannot continue indefinitely and will slow down as the concentration of available building blocks decreases. In summary, this would mean that for an idealized minimal self-replicating system the concentration of the reaction product **T** would show an S-like or sigmoidal shape.

A system in which product inhibition occurs, will not show exponential growth (for exponential growth the kinetic order in replicator *r =* 1) but only sub exponential growth. In many cases *r =* 1/2 and the system is said to obey the square root law of autocatalytic systems [28].

By seeding mixtures with different amounts of preformed templates **T** and measuring the initial rate of template formation, a plot of log(d[**T**]/dt) versus log[**T**] can be constructed. From the slope of this plot the reaction order *r* of the system can be determined [28]. It should however be considered that if the uncatalyzed bimolecular pathway (*r =* 0) also contributes to the formation of **T**, the measured reaction order *r* reflects a weighted average of the catalyzed and uncatalyzed pathways and can therefore have a value smaller than 1, even for cases where the autocatalytic pathway itself would have a reaction order *r =* 1. In such situations computational simulations of the system can provide additional information on the replication processes that are involved [29].

#### 2.4 Achieving exponential replication

**Figure 4:**
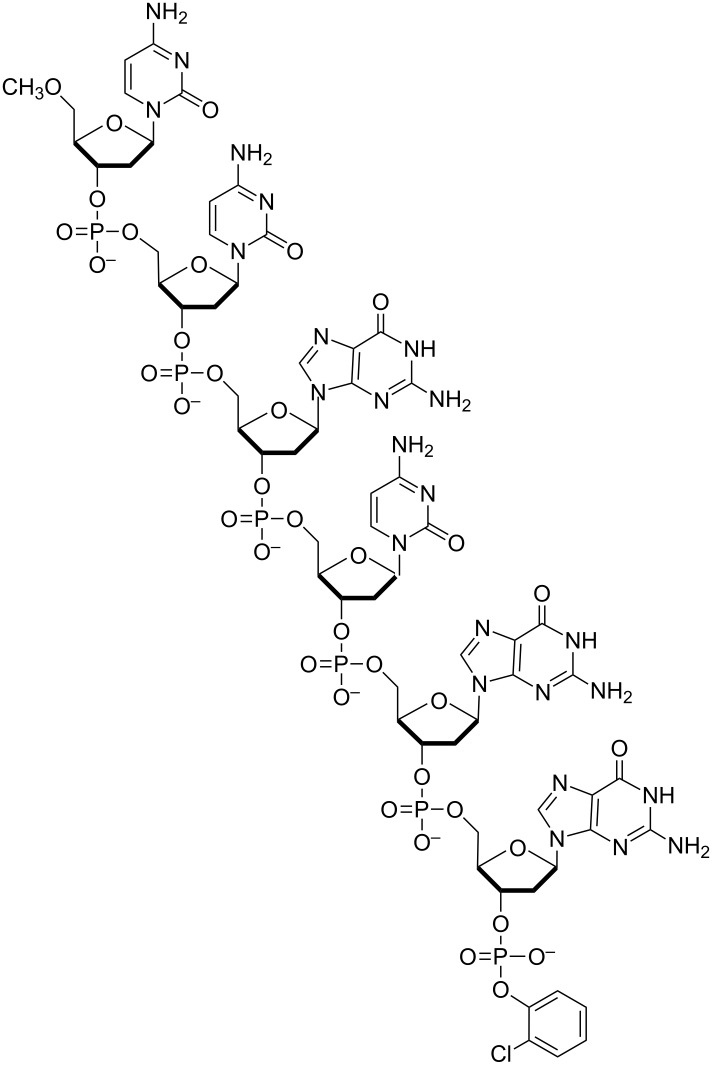
The first oligonucleotide capable of template directed self-replication without the need of enzymes. The depicted hexamer template **T** is formed from two trimer building blocks and catalyzes its own formation. Self-reproduction of this molecule was shown to result in parabolic growth of the template concentration [30].

Pioneering work in the field of non-enzymatic self-replication has been performed by the group of von Kiedrowksi, who was the first to report on a template-directed self-replicating oligonucleotide (Figure 4) [30]. To achieve template-directed self-replication without the aid of enzymes, they used two trinucleotides. Upon activation, these trinucleotides can condense to form a hexamer template molecule **T**, depicted in Figure 4, which catalyzes its own formation. The autocatalytic nature of the reaction was proven by adding small amounts of preformed template molecules to the reaction mixture. Kinetic analysis revealed that the system exhibits parabolic replication (*p =* 1/2). Exponential growth in this system is not obtained due to the high thermodynamic stability of the [**T∙T**] dimer, leading to product inhibition. Although the efficiency of the reported autocatalytic cycle is rather low, it still was a clear demonstration of a template-directed self-replicating system and von Kiedrowski did not fail to recognize the potential of natural selection in such systems.

**Figure 5:**
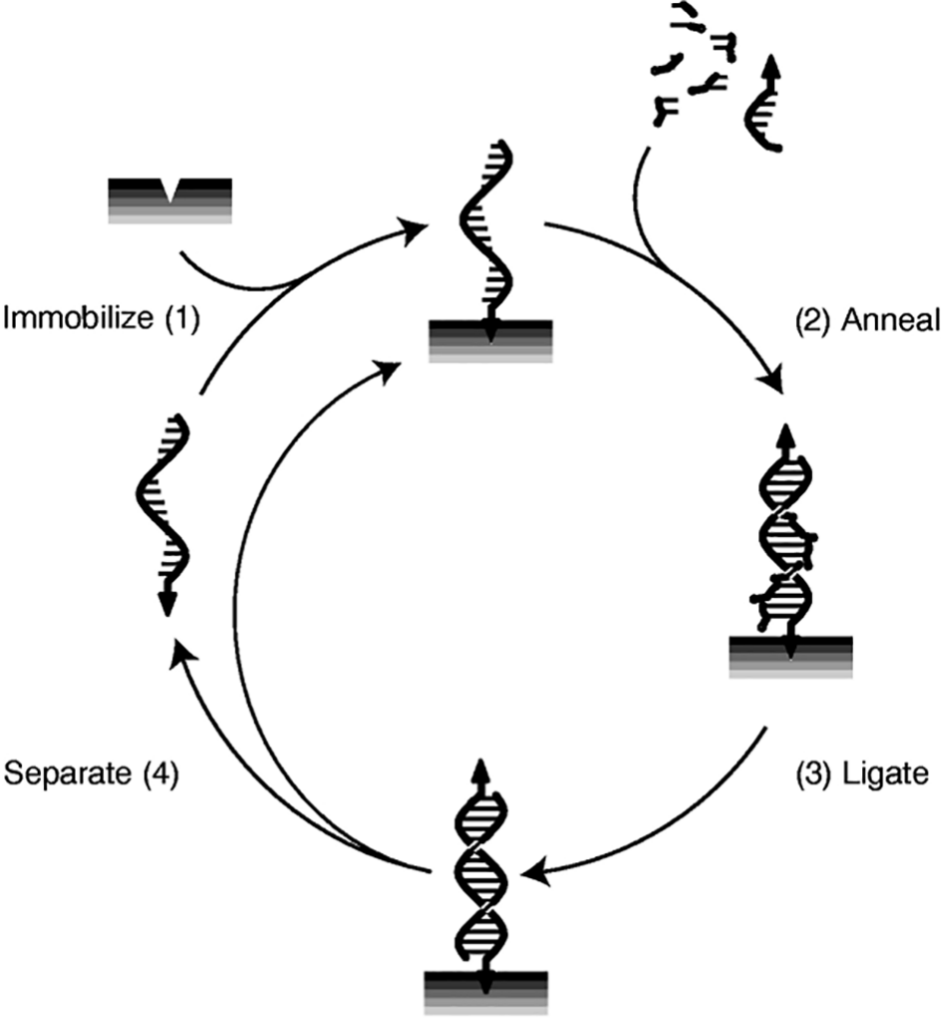
Replication involving the SPREAD technique which prevents product inhibition. (1) A template molecule is immobilized on a solid support and (2, 3) a complementary copy is produced by template-directed replication. Finally the copied strand is (4) released from the template and is in turn immobilized [33].

Later research focused on overcoming the product inhibition problem in order to obtain exponential instead of parabolic growth of the replicators. A successful approach to overcoming product inhibition involves the immobilization of the template molecules by fixing them onto a solid support. This approach was partially inspired by the notion that surfaces of minerals might have played a major role in catalyzing the formation of biopolymers [31–32]. Von Kiedrowski et al. were able to demonstrate exponential growth of oligonucleotides using a method that they gave the eloquent anagram; SPREAD (Surface-Promoted Replication and Exponential Amplification of DNA analogues) [33]. In the SPREAD technique, depicted in Figure 5, an oligonucleotide template strand is immobilized via an irreversible interaction with a solid support. A complementary strand is then produced via the template-directed binding of free nucleotides from the solution. The copied strand is released from the template and is in turn itself immobilized on a solid support, thereby preventing product inhibition via the formation of stable template dimers. As von Kiedrowski and coworkers rightfully notice, this system allows for evolutionary processes to take place. Moreover, such immobilized systems are proposed to be even capable of amplification of mutations. The introduction of mutations can lead to a weaker base pairing between the template molecule and its copy, thus increasing the efficiency of the separation of this particular template duplex.

**Figure 6:**
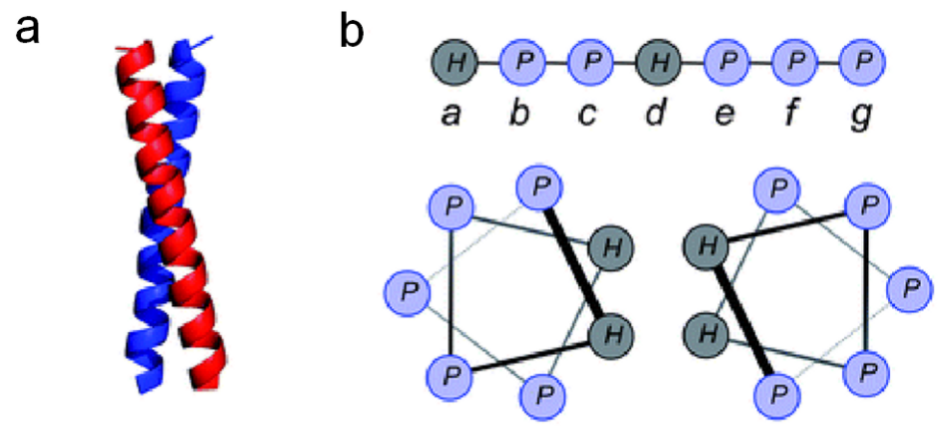
Figure showing (**a**) a coiled coil motif due to hydrophobic interactions between hydrophobic amino acids in the individual helices. (**b**) Helical-wheel diagram showing how the hydrophobic amino acids situated on the a and d sites can interact with each other to form the coiled coil [34].

Considering the proposed abundance of amino acids, it is natural to assume the presence of peptides and oligopeptides under prebiotic conditions. However, initially only very short peptides were produced in experiments under such conditions, raising doubts over their potential role as a precursor of life. When forming α-helices however, longer polypeptides can be stabilized by the formation of coiled-coil motifs as in Figure 6. If every a and d position of each individual helix is occupied by a hydrophobic amino acid, the helices can intertwine and bury their hydrophobic side groups into each other. This hydrophobic interaction that drives the formation of coiled-coil motifs can be further enhanced by electrostatic interactions between amino acids residing on the c and g positions of the α-helices.

**Figure 7:**
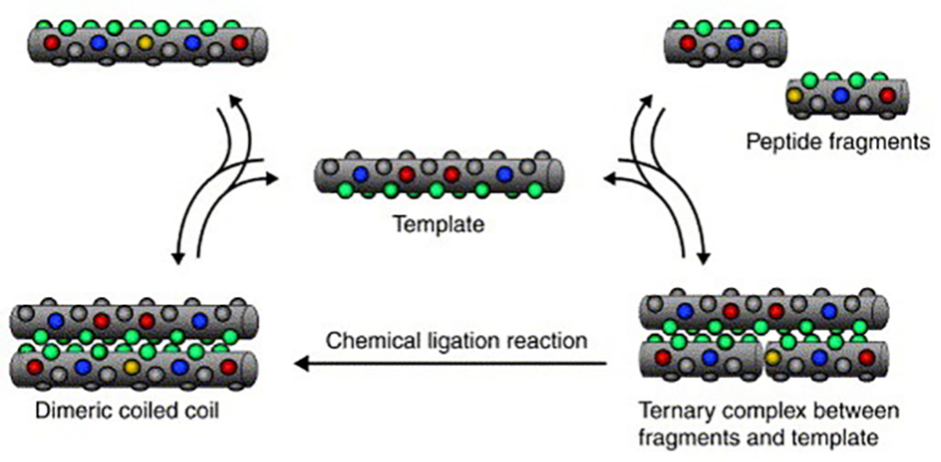
Self-replication of a helical peptide. Molecular recognition leads to the formation of a stable coiled coil structure from smaller peptide fragments. Depending on the length and stability of the coiled coil structure, the template-duplex dissociates in the original template and a copy [37].

Ghadiri et al. showed that such coiled-coil peptides are capable of self-replication [35]. As depicted in Figure 7, helical polypeptides can act as a template for shorter peptide fragments by means of molecular recognition. The peptide building blocks again are ligated, resulting in the formation of a template duplex with a coiled-coil motif. When separated from the original template, a copy of the template is obtained. Initially these replicating systems were reported to show only parabolic growth, because of the very high stability of the coiled-coil structure. This problem was later addressed by Issac and coworkers by reducing the length of the template molecule, which led to a decreased stability of the template duplex. Using this approach they obtained near exponential growth of the template concentration of *p* = 0,91 [36–37]. The above examples all illustrate that, while not trivial, it is indeed possible to obtain self-replicating behavior in the absence of enzymes. While this marks a significant contribution to our understanding of the early stages of the transition from chemistry to biology, it does not directly explain the emergence of the RNA and DNA dominated world as we know it, which would probably have required open-ended evolution.

### 3 Evolutionary dynamics of replicators

#### 3.1 Enzyme mediated replication

Iconic early experiments aiming to achieve Darwinian type evolution in a chemical system were performed by Spiegelman et al. in 1967 [3]. RNA replicase and a small input of genomic RNA were successfully isolated from the bacteriophage Qβ. The RNA molecules in this system are replicated by an RNA replicase enzyme. By successive rounds of amplification and selection, selection pressure was introduced to the system by favoring fast reproducing entities of the genomic RNA. Since shorter sequences are being replicated at a higher rate than longer sequences, shortened mutants are favored over longer sequences. This eventually led to a strong decrease in the genome size of the RNA molecules. However, this result is not as trivial as it may seem at first sight, since it is of vital importance that the mutant species do not lose their ability to be replicated, indicating that only specific parts of the genome that are not needed for recognition by the polymerase were deleted. Although the RNA molecules involved are not self-replicating but are replicated by the RNA replicase, the study still marks a starting point in the field of in vitro evolution. Later, Braun et al. managed to apply a selection pressure that favors the replication of long DNA sequences over short strands by creating heat gradients in pores that act as a thermal trap [38]. The thermal traps selectively retain longer DNA sequences, thereby effectively overcoming the inherent advantage of the replication of short sequences.

#### 3.2 Dynamics of self-replicators

Ashkenasy recently reported a peptide based synthetic autocatalytic network that shows two significantly distinct steady states depending on the history of the system [39]. Depending on the initial concentration of replicator molecules provided to the system, the system will reach either a low or a high steady state replicator concentration. Switching between these two states can be achieved by applying external stimuli in the form of heat or the addition of decomposing agents. The switchable behavior and memory of such a self-replicating system constitute an exciting step, moving systems of self-replicating molecules away from equilibrium, with potential impact on evolutionary behavior [40].

Another interesting dynamic emergent property of self-replicating systems was demonstrated by Philp and coworkers [41]. They showed how self-replicating molecules can create a reaction-diffusion front when seeded to a homogeneous mixture of building blocks. Dynamically evolving out-of-equilibrium environments like these could enable interesting behavior of replicators that is not achievable in homogenous reaction mixtures. It will be very exciting to observe the evolutionary behavior of mixtures of replicators in such spatially resolved environments.

#### 3.3 RNA self-replication

Owing to the importance of RNA in viral species and in the origin of life, evolution experiments are most often performed using RNA molecules or closely related derivatives. In fact, it has become possible to perform natural selection on oligonucleotides by iterative amplification and selection processes using a technique called systematic evolution of ligands by exponential enrichment, or SELEX. In SELEX, a library of DNA and RNA sequences is exposed to a certain target. In multiple selection rounds the binding species are selected and amplified, while the non-binding DNA and RNA molecules are disposed of. In this way molecules are evolved based on their ability to bind to a specific target [42–43].

However, these in vitro evolution experiments all exploit RNA-based enzymes (ribozymes) or proteins in their replication process to obtain exponential growth and are consequently not self-replicating. Efforts have been made to obtain in vitro evolution of RNA in the absence of any enzymes. Unfortunately, the demonstration of multiple cycles of non-enzymatic RNA replication in a test tube is troubled by the fact that the RNA duplex that is formed upon replication is quite stable and can have dissociation temperatures as high as 90 °C [44]. Without an enzyme that separates the newly created strands, this stability would lead to product inhibition, halting the self-replication process.

#### 3.4 Cross-catalyzing RNA replicators

**Figure 8:**
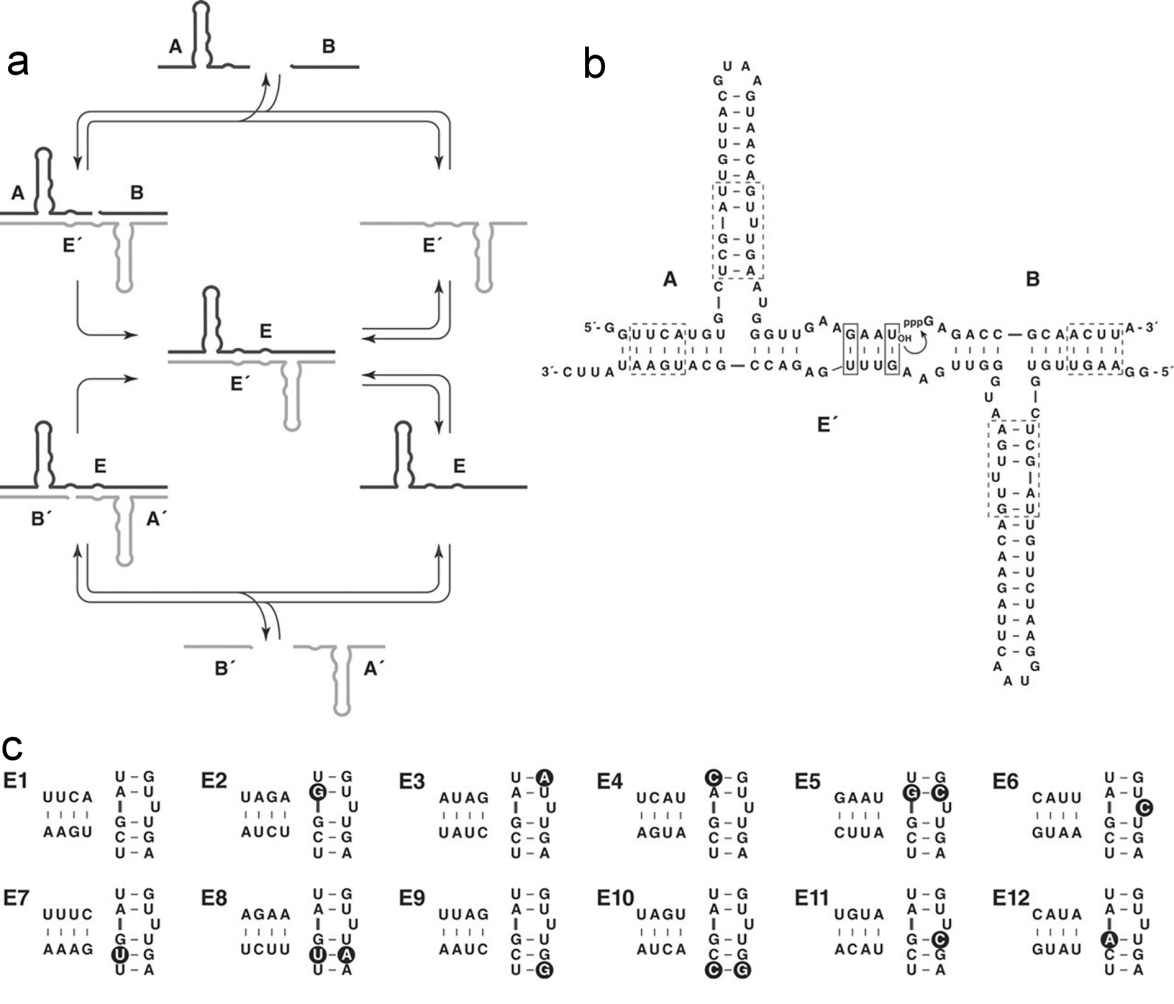
(a) Cross-catalyzed replication of template molecules **E** and **E’** from their building blocks **A^(^’^)^** and **B^(^’^)^**. (b) Secondary structure of the template duplex. The curved arrow denotes the site of ligation of the building blocks, the dashed boxes include the sequences to be mutated and the solid lines indicate the sites of the **G∙U** base pairs inducing the wobble that enhances catalytic activity. (c) The altered sequences of the 12 different template molecules, the **E’** molecules have complementary sequences in the base pairing part (horizontal) and identical sequences in the catalytic part (vertical). Dark circles denote the differences relative to **E1** [45].

Joyce and Lincoln showed, however, that a system of two RNA enzymes can catalyze each other’s synthesis from a mixture of four different building blocks via template-directed reciprocal replication [45]. The RNA ligase molecule **E** can bind two oligonucleotide building blocks **A’** and **B’** and promote their ligation to form the ligase **E’**. The newly formed ligase **E’** can then in turn promote the formation of **E**, as depicted in Figure 8a. But this cross-catalytic reaction typically occurs at a very slow rate. In order to enhance this rate, enzymatic in vitro evolution of the RNA molecules was performed in order to obtain a set of fast replicating species.

It was found from in vitro evolution experiments that the introduction of **G∙U** base pairs close to the site of ligation leads to enhanced cross-catalytic activity. Figure 8b shows the sequence and secondary structure of the **A∙B∙E’** complex. The site of ligation is indicated by the curved arrow and the **G∙U** pairs that are depicted in a solid box induce a wobble in the sequence that results in the enhanced catalytic activity. When this wobble is installed in both enzymes of the cross-catalytic set, exponential growth of the system can be achieved over multiple cycles. With an exponential replicator in hand, Darwinian evolution in a cross-catalytic system lies within reach. To study this, Joyce and his team prepared 12 pairs of cross-catalytic enzymes and their corresponding building blocks, that have alteration in parts of the sequence denoted by the dashed line in Figure 8b. The different pairs are denoted as **E1** to **E12** and are shown in Figure 8c. It is important to note that mutations between enzymes are such that the stability of the ligase duplex due to base pairing is not altered, but only the catalytic activity and replication rate are affected. All these enzymes were shown to cross-replicate, with the **E1** pair showing the highest rate of replication.

**Figure 9:**
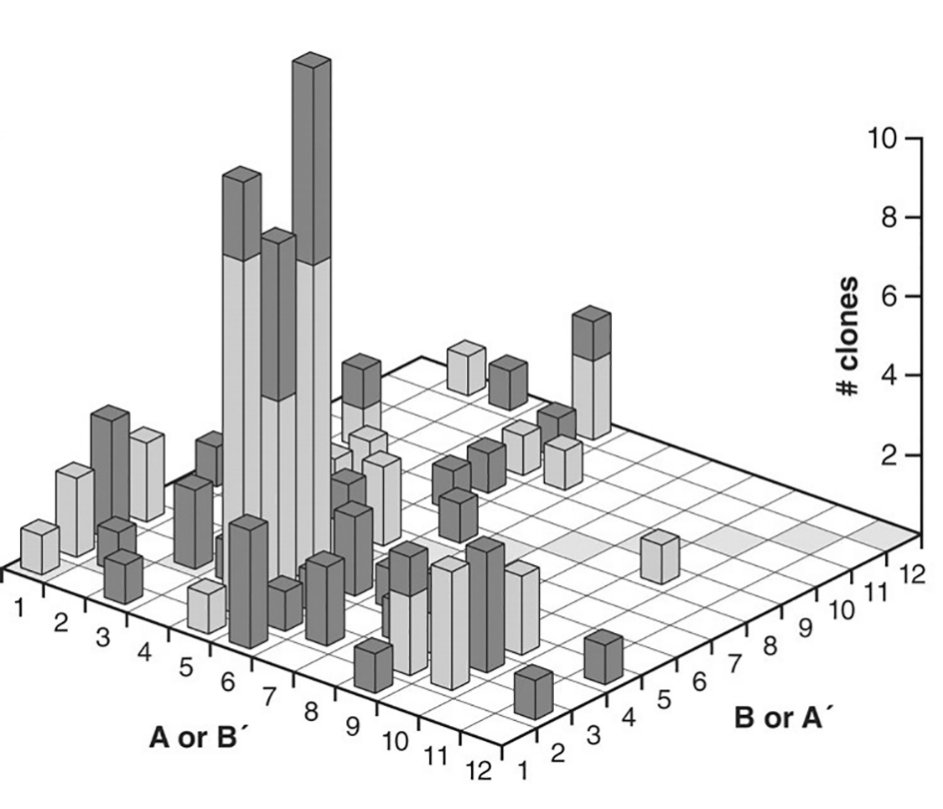
Distribution of the species present in the reaction mixture after 20 serial transfers. **E** and **E’** molecules are represented by the dark and light shaded bars, respectively. Note how certain species have come to dominate the population, particularly **A**5**B**3. Moreover, only 7 molecules were found to be paired to their original partner (corresponding to the shaded diagonal) [45].

A serial transfer experiment was performed on a mixture that contained the 12 different enzyme pairs and their corresponding 48 building blocks (**A1**, **A1’**, **B1**, and **B1’** for pair **E1**, for example). In such a serial transfer experiment a small percentage, in this case 5%, is transferred to a new reaction mixture after a replication round took place. This effectively eliminates the slow replicators that are only present in small quantities in the mixture so that they tend to go extinct. The transferred replicators, however, are presented with a fresh batch of building blocks and can continue to replicate. By doing this for multiple rounds, large amplification factors can be achieved. For this experiment it is important to realize that **A1** does not necessarily have to be ligated to **B1**, but that it can ligate to any of the other **B**-type building blocks, although they may be mismatched to the template. This freedom of recombination leads to 132 possible combinations of building blocks. After 20 successive transfers a 1025-fold amplification was reached. A sample of 100 of these clones contained only 7 non-recombinant clones, whereas the rest were all ligated to building blocks that were not their original partners. Figure 9 shows the distribution of different **E** (dark columns) and **E’** (light columns) enzymes in the final sample. This result shows how fitter replicators can come to dominate the population after several rounds of amplification. Fitness of the molecules depends in this case on their ability to perform cross-catalytic replication with other molecules.

This study by Joyce et al. demonstrates how selection pressure can lead to certain replicators dominating a population in a cross-catalytic replication process. However, the environmental conditions in this experiment are static and the system lacks open-endedness because the number of building blocks that is provided to the system restricts the total diversity of the newly formed species, in this case 12 × 12 different possible replicators. This will cause the system to reach a steady state in which no novel forms of the replicator can be explored anymore.

#### 3.5 Cooperative catalytic system

The concept of such a cross-replicating system can be readily extended to higher order systems, involving three, four or even more components. Eventually, one could envision an entire network of cross-replicating molecules. Lehman et al. showed that a mixture of relatively short RNA segments can self-assemble to form self-replicating ribozymes [46]. These ribozymes in turn gave rise to spontaneous formation of cooperative networks that were shown to grow faster than the autocatalytic replication rate of the individual ribozymes. Moreover, cooperative systems are generally more stable towards parasites then autocatalytic self-replicators and are, in principle, able to gain in complexity [46–47].

**Figure 10:**
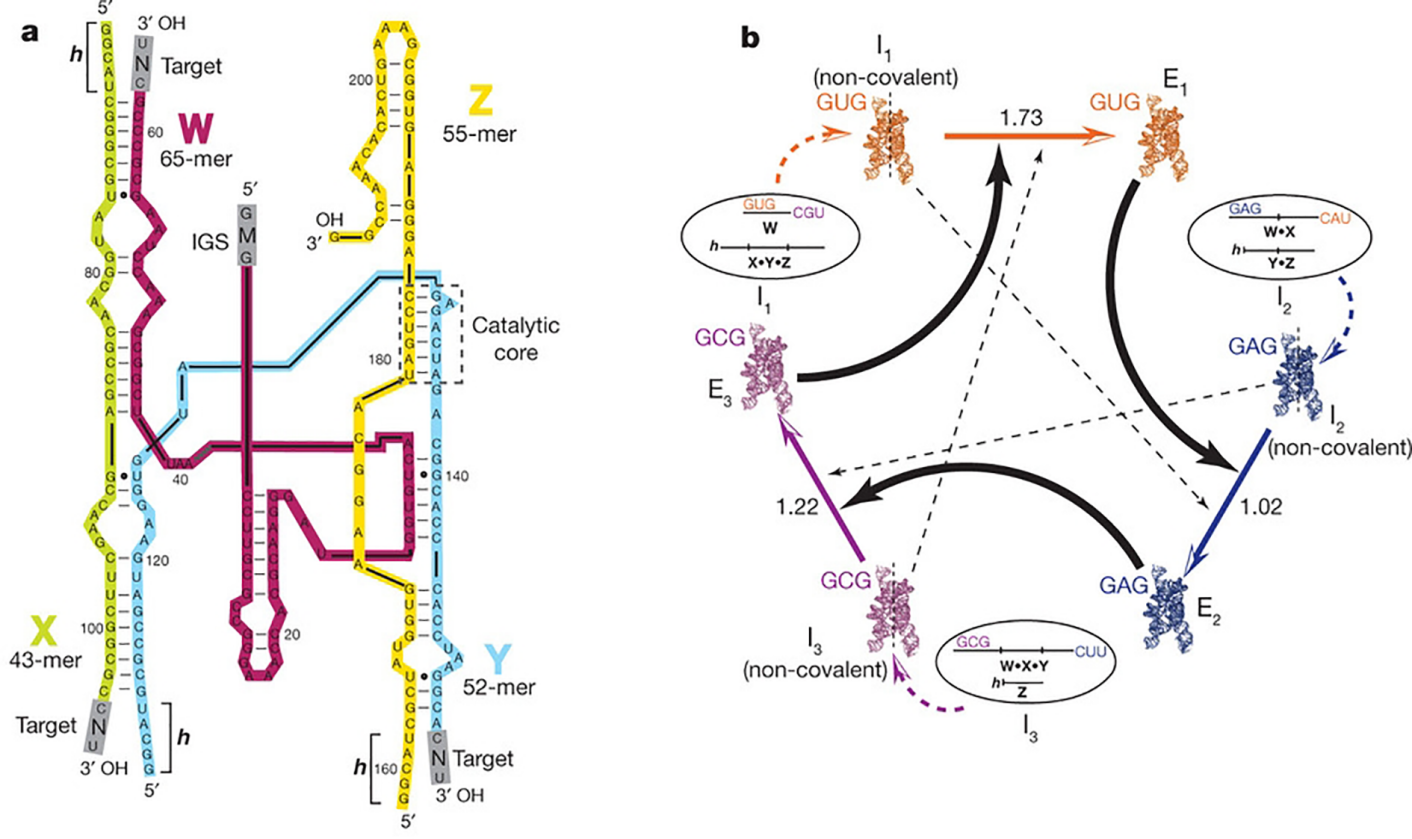
(a) Secondary structure of the *Azoarcus* ribozyme consisting of four different strands of RNA, W, X, Y, and Z. Self-replication is mediated by recognition of the target sites by the IGS (grey boxes), leading to ligation of the strands. The dashed line indicates the catalytic core of the resulting ribozyme. (b) Cooperative replicating system. The formation of the covalent ribozyme **E3** from the non-covalent **I3** complex is catalyzed by **E2** ribozyme. The formation of this **E2** is in turn catalyzed by **E1**, which is catalyzed by **E3**, resulting in a cyclic dependence. Numbers above the arrows denote the advantage of cooperativity [46].

In the study a ribozyme of around 200 nucleotides called *Azoarcus* was used. This ribozyme is made from four different RNA strands (W, X, Y and Z) that can self-assemble covalently in an autocatalytic manner, as depicted in Figure 10a. The effectiveness of this self-replication process depends on the ability of the internal guide strand (IGS) to recognize its target. To form a cooperative set, the *Azoarcus* ribozyme was fragmented in two different pieces in three different ways, creating three different pairs I1, I2 and I3 which are shown encircled in Figure 10b. Furthermore, the target and IGS sequences were altered such that autocatalytic self-replication is minimized. The sequence was, however, chosen such that the IGS of one pair is matched to the target sites of the next pair. In this way one ribozyme, say **E1**, can catalyze the formation of the next ribozyme, **E2**, from its non-covalently bound building blocks **I2**. This ribozyme can in turn catalyze the formation of **E3** from its building block and finally, to close the cycle, **E3** can catalyze the formation of the **E1** ribozyme. This cooperative system is depicted in Figure 10b and it was observed that a mixture containing all three pairs resulted in a much higher yield of full-length RNA (a factor 125) than obtained from the sum of the isolated pairs, proving that the system replicates in a cooperative manner.

**Figure 11:**
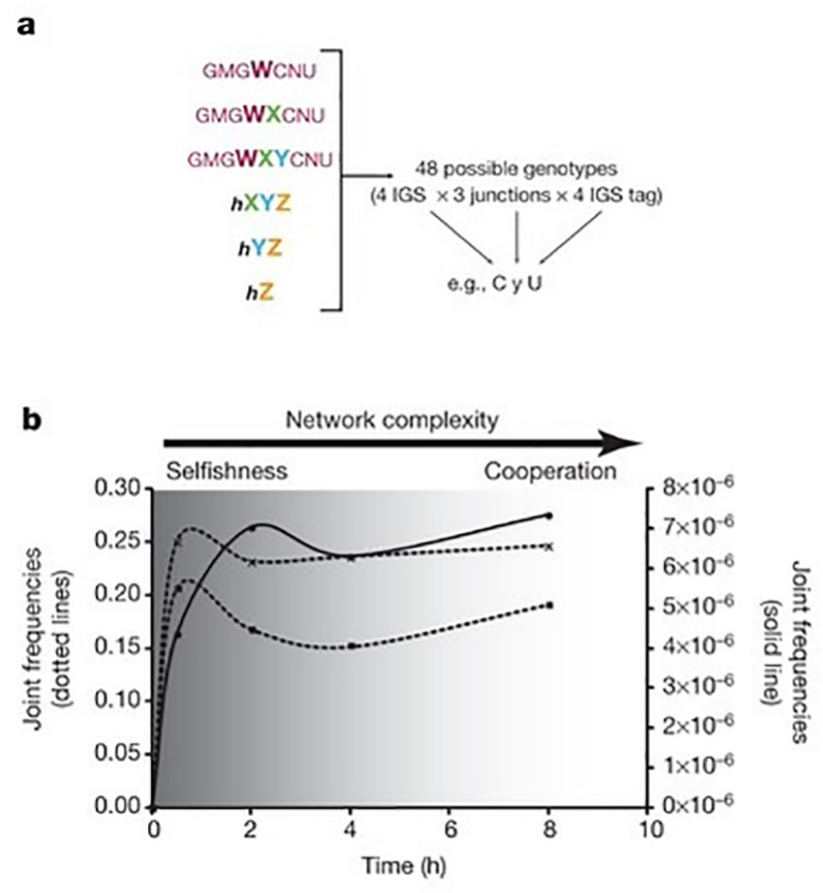
(a) The different combinations of IGS strands, tags and break junction give rise to a total of 48 different pairs. (b) Graph showing the frequency of autocatalytic replicators (dashed, crosses), the two-membered cycles (dashed, dots; ×10) and the three-membered cycles (solid; ×10.000) over time. Note the emergence of the more complex three-membered cycles at later times [46].

Interestingly, it was shown that in isolation the autocatalytic replicators (with the IGS programmed to recognize itself) replicated faster than the cross-catalytic system, whereas in a mixture of all different components the cooperative network grows faster than the selfishly replicating molecules. However, this result was obtained using deliberately designed pairs with specific targets. Behavior becomes a lot more fascinating when one of the nucleotides of the IGS (M) and target sites (N) is randomized, creating a mixture of 48 matched and unmatched pairs in total, as schematically depicted in Figure 11a. After incubation of all six sets of Figure 11a for several hours, all of these 48 possible sequences were indeed found in the mixture. Initially the replication is dominated by autocatalytic cycles in which N and M are complimentary. This initial rise of the autocatalytic replicators is depicted in Figure 11b by the dashed line with crosses, the contribution of the two-membered cycles is depicted by the dashed lines with dots (depicted value ×10). At later times a transition to the more complex three-membered cycles was observed as witnessed by the rise of the solid line (×10.000). After 8 hours, it was observed that replication occurs increasingly via cooperative cycles and that all genotypes contribute increasingly with time. This result shows how an initially autocatalytic cycle can give rise to increasingly complex systems of cooperative replication over time. Interestingly, the overall replication efficiency of the randomized multi-component network exceeded that of the engineered 3-component network in Figure 10b.

To better mimic prebiotic conditions in which iterations over multiple generations would have occurred, a serial transfer experiment using the same set of replicators was also performed. In this experiment an aliquot of the reaction mixture is transferred to a new flask with building blocks every hour, so that the more stable and fast replicating molecules and networks are favored. Again a transition from autocatalytic cycles to more complex systems was observed.

Such cooperative systems are capable of complexification and natural selection and can therefore be of importance in bridging the gap between replication of simple short RNA molecules from nucleotide building blocks and the formation of more complex ribozymes. The observed cooperative behavior relies on recognition strands and tags, so that it will only play a role for the assembly of intermediate-sized oligonucleotides. Small oligonucleotides would likely still replicate more efficiently via auto or cross catalytic cycles. At a certain length scale the formation of cooperative systems becomes favorable and these mechanisms might take over the replication process, allowing for complexification and diversification of the system. However, since the replication of each member is dependent on one or more other members of the system, the members should all be in close proximity to each other in order to obtain a stable system. This requires high concentrations of the reaction mixture, which is of course readily achieved in the laboratory but is probably less likely under prebiotic conditions. In order to increase the concentration of replicators locally, a specialized compartmentalization should act in concert with the cooperative replication system. How such compartmentalization might occur is another topic entirely and beyond the scope of this review, but it is proposed that compartmentalization can actually aid in the evolution of replicating molecules [48–50].

#### 3.6 Diversification of self-replicators

**Figure 12:**
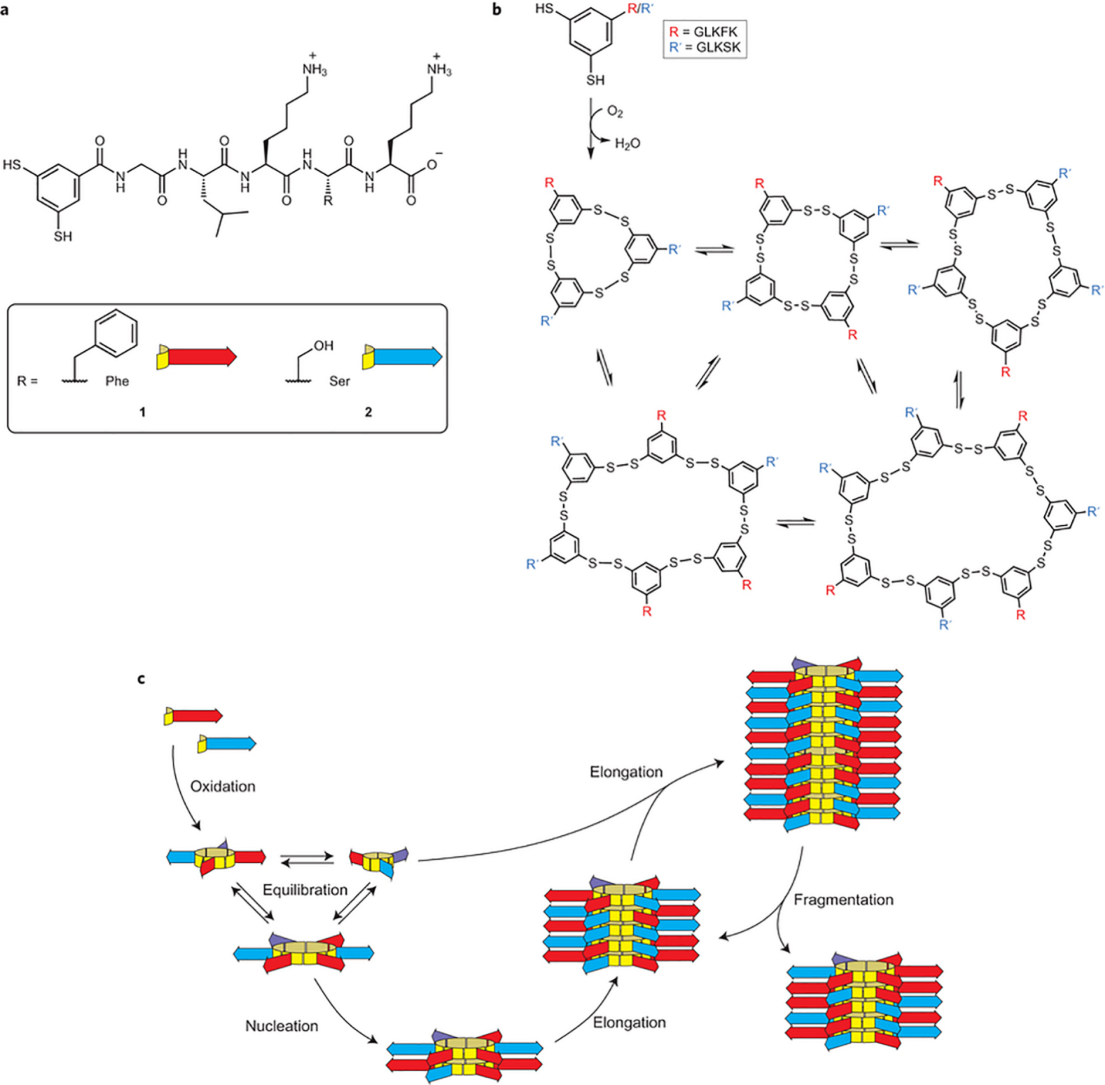
Figure depicting (a) building blocks consisting of a peptide attached to an aromatic ring. Building blocks **1** and **2** differ only in the nature of the penultimate amino acid. (b) The building blocks can form macrocycles of different sizes upon oxidation, which can exchange building blocks with each other. (c) Schematic representation showing how building blocks oxidize to form macrocycles that in turn form stacks due to β-sheet formation. Stacks grow from their ends and fragment upon agitation, leading to more fiber ends and faster growth [52].

Other types of molecules than RNA that are capable of self-replication and information storage are showing interesting results in the study of open-ended evolution and the synthesis of life as well [51]. Recently, we have demonstrated a self-replicating system involving peptides capable of diversification using a systems chemistry approach [52]. Following the discovery of an exponentially growing self-replicating system [53], we used two building blocks, **1** and **2**, to form a dynamic combinational library (DCL) of self-replicating molecules. These building blocks consist of an aromatic core that is functionalized with two thiol groups and a peptide chain (Figure 12a). Building block **1** and **2** are very closely related to each other and differ only in a single amino acid of the peptide chain. These peptide building blocks can then be oxidized to form macrocycles of different sizes as depicted in Figure 12b. The design of the peptide chains is such that self-assembly of the chains into parallel β-sheets is promoted, which in turn leads to the formation of stacks of macrocycles as shown in Figure 12c. Growth of these stacks occurs exclusively via the ends of the fibers and it is therefore not surprising that the reaction rate is strongly dependent on the amount of fibers present in the mixture. As soon as a fiber reaches a critical length it can fragment when mechanically agitated. When fragmentation occurs, the number of available fiber ends is doubled, leading to an exponential self-replication.

In previous work it was already shown that the less hydrophobic building block **2** tends to form larger octameric macrocycles than the more hydrophobic building block **1** which forms hexamers [54]. This is reasonable, since a weaker hydrophobic interaction provided by **2** would need more individual interactions in order to achieve the same stability as a more hydrophobic counterpart **1**.

**Figure 13:**
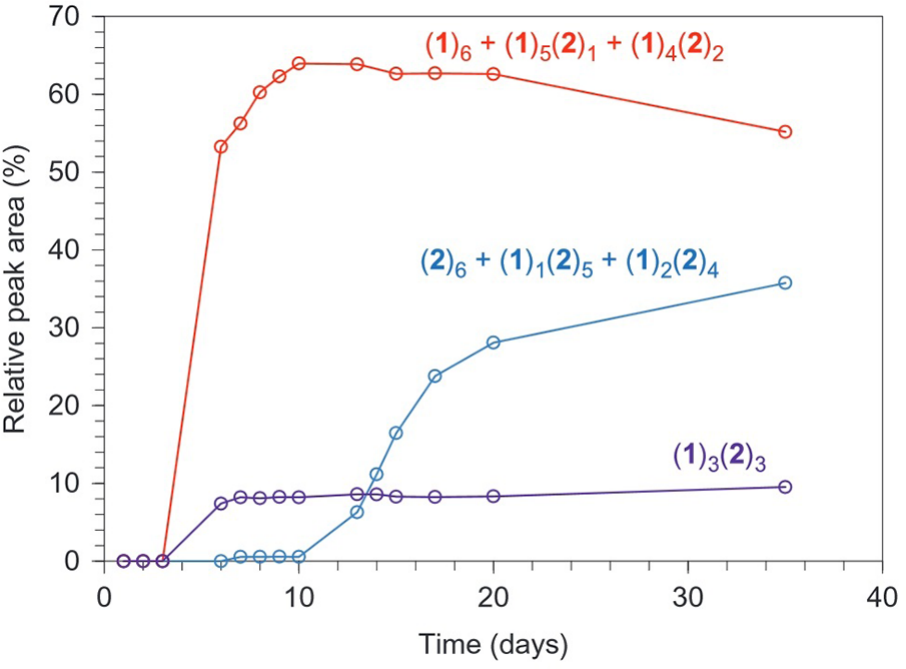
Plot showing the relative concentrations of set I (red), set II (blue) and the link between them; (1)_3_(2)_3_ (purple). Note how at first only set I is present in the mixture, while at some point this set gives rise to the descendant set II. [52]

By using a mixture containing two different building blocks instead of one, the replicators can potentially undergo mutation by incorporating a different building block into their structures. A mixture with equal concentrations of both building blocks was prepared and monitored over the course of 35 days. Initially a complex mixture of four different trimers and five different tetramers was observed. After some days, however, a set of hexamers which was enriched in building block **1** arose in the mixture (set I) as shown by the red line in Figure 13. As the emergence of set I depletes the mixture from building block **1** the environmental conditions are essentially changed up to the point where a second set of hexamers arises which is rich in building block **2**.

It was shown that set I is the ancestor of set II. When macrocycles that are rich in building block **2** are exposed at the fiber ends of set I, they act as a template for the formation of members of set II. Indeed, significant amounts of set II members only form when a seed of set I is present that contains **2**-enriched members. Set I is therefore able to transfer information about its macrocyclic size to set II. This process bears a crude resemblance to how species originate in biology.

## Conclusion

Self-replicating molecules have been remarkably hard to develop and after 30 years of research there are still only a handful of efficient self-replicators. Achieving Darwinian evolution with these systems has proven even more challenging. The evolutionary potential of many self-replicating molecules is limited due to the fact that it is difficult to achieve exponential growth of the replicator. Factors limiting the efficiency of the self-replication process are the presence of non-autocatalytic pathways and product inhibition. Methods aiming to minimize the effect of product inhibition, like the SPREAD technique and the destabilization of template-duplexes, have successfully been developed to allow for exponential growth of some simple replicators. Also mechanical forces may be utilized to break up larger assemblies of self-replicating molecules and liberate the assembly edges or fiber ends that promote replication.

The most impressive progress with respect to Darwinian evolution has been achieved with RNA-based cross-replicators. In serial transfer experiments changes in replicator populations were observed that were not immediately predictable and that favored the most efficient replicators or networks of cooperating replicators. What these systems have not (yet) shown is the emergence of new functions that contribute to the dynamic kinetic stability of the replicators.

The true challenge of any in vitro evolution experiment lies in the realization of a system that has the capability to undergo open-ended evolution. Such systems can diversify and increase in complexity and invent new functions indefinitely. Until now, chemical systems that show evolutionary behavior have involved relatively simple replicators that only had access to a very limited structural space of possible mutations. This rapidly causes the system to be incapable of exploring new structures and the development of novelty will stagnate. An additional limitation of simple replicators is the strong relation between their genotype and phenotype. This lack of dichotomy causes the mechanisms of mutation and natural selection to couple to each another, hampering the evolvability of the systems. It is far from trivial to design a system that is simple enough to be capable of exponential replication and has a large structural space of mutations at the same time. Yet a push in this direction is probably needed, expanding the structural space available for existing replicators to explore, enabling them to discover new functions, one of which might eventually be the decoupling between genotype and phenotype, which would allow the system to explore a dramatically larger structural and functional space.

Besides these issues concerning the design of replicators, it is still not studied in detail how the environment of the replicators can interact with the evolutionary process. Can environmental conditions like acidity or temperature, for instance, be an incentive towards the development of novel functionalities in the replicators? And how is the notion of death introduced in an experiment in which the researcher does not actively intervene with the system through, for example, serial dilution? In any true open-ended system replicators interact with the environment on their own account and are not steered by the experimenter to a significant extent.

Thus, the challenge is now to design systems of self- or cross-replicating molecules that can access and evolve into a vast structural and functional space and facilitate, by appropriate design of building blocks and experimental conditions, the invention of new functions and thereby achieve open-ended evolution.

## References

[1] Kauffmann S A (1995). At Home in the Universe.

[2] Darwin C (1871). On the Origin of Species.

[3] Mills D R, Peterson R L, Spiegelman S (1967). Proc Natl Acad Sci U S A.

[4] Joyce G F (2007). Angew Chem, Int Ed.

[5] Szathmary E, Gladkih I (1989). J Theor Biol.

[6] Lifson S, Lifson H (2001). J Theor Biol.

[7] Joyce G F (2004). Annu Rev Biochem.

[8] Eigen M, McCaskill J, Schuster P (1988). J Phys Chem.

[9] Ruiz-Mirazo K, Briones C, De la Escosura A (2014). Chem Rev.

[10] Eigen M (2002). Proc Natl Acad Sci U S A.

[11] Arenas C D, Lehman N (2010). BMC Evol Biol.

[12] Biebricher C K, Eigen M (2005). Virus Res.

[13] Crotty S, Cameron C E, Andino R (2001). Proc Natl Acad Sci U S A.

[14] Pascal R, Pross A (2017). Synlett.

[15] Ruiz-Mirazo K, Pereto J, Moreno A (2004). Origins Life Evol Biospheres.

[R16] 16Taylor, T. *European conference on artificial life 2015,* York, UK, July, 2015.

[17] Moreno A, Ruiz-Mirazo K (2009). Biol Philos.

[18] Taylor T J (1999). From Artificial Evolution to Artificial Life.

[19] Von Neumann J, Burks A W (1966). IEEE Trans Neural Networks.

[20] Ruiz-Mirazo K, Umerez J, Moreno A (2008). Biol Philos.

[21] Szathmáry E, Maynard Smith J (1995). Nature.

[R22] 22Crutchfield J.P.; Schuster, P. Genotype and phenotype. In *Evolutionary Dynamics: Exploring the Interplay of Selection, Accident, Neutrality, and Function*, Oxford University Press: Oxford, U.K., 2003; pp 164-169.

[23] Taylor T, Bedau M, Channon A, Ackley D, Banzhaf W, Beslon G, Dolson E, Froes T, Hickinbotham S, Ikegami T (2016). Artif Life.

[24] Vidonne A, Philp D (2009). Eur J Org Chem.

[25] Bag B J, Von Kiedrowski G (2009). Pure Appl Chem.

[26] Bissette A J, Fletcher S L (2013). Angew Chem, Int Ed.

[27] Reinhoudt D N, Rudkevich D M, De Jong F (1996). J Am Chem Soc.

[28] Von Kiedrowski G (1993). Bioorg Chem Front.

[29] Coulomb-Delsuc M, Mattia E, Sadownik J W, Otto S (2015). Nat Commun.

[30] Von Kiedrowski G (1986). Angew Chem, Int Ed Engl.

[31] Ferris J P, Hill A R, Liu R, Orgel L E (1996). Nature.

[32] Ferris J P, Ertem G (1992). Science.

[33] Luther A, Brandsch R, Von Kiedrowski G (1998). Nature.

[34] Armstrong C T, Boyle A L, Bromley E H C, Mahmoud Z N, Smith L, Thomson A R, Woolfson D N (2009). Faraday Discuss.

[35] Lee D H, Granja J R, Martinez J A, Severin K, Ghadiri M R (1996). Nature.

[36] Issac R, Chmielewski J (2002). J Am Chem Soc.

[37] Issac R, Ham Y W, Chmielewski J (2001). Curr Opin Struct Biol.

[38] Kreysing M, Keil L, Lanzmich S, Braun D (2015). Nat Chem.

[39] Mukherjee R, Cohen-Luria R, Wagner N, Ashkenasy G (2015). Angew Chem, Int Ed.

[40] Decker P (1973). Nature (London).

[41] Bottero I, Huck J, Kosikova T, Philp D (2016). J Am Chem Soc.

[42] Keefe A D, Pai S, Ellington A (2010). Nat Rev Drug Discovery.

[43] Mahlknecht G, Maron R, Mancini M, Schechter B, Sela M, Yarden Y (2013). Proc Natl Acad Sci U S A.

[44] Hernandez A R, Piccirilli J A (2013). Nat Chem.

[45] Lincoln T A, Joyce G F (2009). Science.

[46] Vaidya N, Manapat M L, Chen I A, Xulvi-Brunet R, Hayden E J, Lehman N (2012). Nature.

[47] Higgs P G, Lehman N (2015). Nat Rev Genet.

[48] Ghadessy F J, Ong J L, Holliger P (2001). Proc Natl Acad Sci U S A.

[49] Szostak J W, Bartel D P, Luisi P L (2001). Nature.

[50] Matsumura S, Kun A, Ryckelynck M, Coldren F, Szilágyi A, Jossinet F, Rick C, Nghe P, Szathmáry E, Griffiths A D (2016). Science.

[51] Pinheiro V B, Taylor A I, Cozens C, Abramov M, Renders M, Zhang S, Chaput J C, Wengel J, Peak-Chew S Y, McLaughlin S H (2012). Science.

[52] Sadownik J W, Mattia E, Nowak P, Otto S (2016). Nat Chem.

[53] Carnall J M A, Waudby C A, Belenguer A M, Stuart M C A, Peyralans J J P, Otto S (2010). Science.

[54] Malakoutikhah M, Peyralans J J P, Colomb-Delsuc M, Fanlo-Virgós H, Stuart M C A, Otto S (2013). J Am Chem Soc.

